# Transcriptomic Analyses of Ovarian Clear Cell Carcinoma Spheroids Reveal Distinct Proliferative Phenotypes and Therapeutic Vulnerabilities

**DOI:** 10.3390/cells14110785

**Published:** 2025-05-27

**Authors:** Bart Kolendowski, Sylvia Cheng, Yudith Ramos Valdes, Trevor G. Shepherd, Gabriel E. DiMattia

**Affiliations:** 1 Mary & John Knight Translational Ovarian Cancer Research Unit, Verspeeten Family Cancer Centre, London, ON N6A 4L6, Canada; bkolendo@uwo.ca (B.K.); sche243@uwo.ca (S.C.); yudithramos@yahoo.es (Y.R.V.); tshephe6@uwo.ca (T.G.S.); 2 London Health Sciences Centre Research Institute, London, ON N6A 4L6, Canada; 3 Department of Biochemistry, Western University, London, ON N6A 4L6, Canada; 4 Department of Oncology, Western University, London, ON N6A 4L6, Canada; 5 Department of Anatomy & Cell Biology, Western University, London, ON N6A 4L6, Canada; 6 Department of Obstetrics & Gynaecology, Western University, London, ON N6A 4L6, Canada

**Keywords:** clear cell, ovarian, cancer, cell line, G2/M, spheroid, dormancy, AZD1775

## Abstract

Cancer cell spheroids autonomously form in the ascites fluid and are considered a conduit for epithelial ovarian cancer metastasis within the peritoneal cavity. Spheroids are homotypic, avascular 3D structures that acquire resistance to anoikis to remain viable after cellular detachment. We used in vitro spheroid model systems to interrogate pathways critical for spheroid cell proliferation, distinct from those driving monolayer cancer cell proliferation. Using the 105C and KOC-7c human ovarian clear cell carcinoma (OCCC) cell lines, which have distinct proliferative phenotypes as spheroids but the same prototypical OCCC gene mutation profile of constitutively activated AKT signaling with the loss of ARID1A, we revealed therapeutic targets that efficiently kill cells in spheroids. RNA-seq analyses compared the transcriptome of 3-day monolayer and spheroid cells from these lines and identified the characteristics of dormant spheroid cell survival, which included the G2/M checkpoint, autophagy, and other stress pathways induced in 105C spheroids, in sharp contrast to the proliferating spheroid cells of the KOC-7c cell line. Next, we assessed levels of various G2/M checkpoint regulators and found a consistent reduction in steady-state levels of checkpoint regulators in dormant spheroid cells, but not proliferative spheroids. Our studies showed that proliferative spheroid cells were sensitive to Wee1 inhibition by AZD1775, but the dormant spheroid cells showed a degree of resistance to AZD1775, both in terms of EC50 values and spheroid reattachment abilities. Thus, we identified biomarkers of dormant spheroids, including the G2/M checkpoint regulators Wee1, Cdc25c, and PLK1, and showed that, when compared to proliferating spheroid cells, the transcriptome of dormant OCCC spheroids is a source of therapeutic targets.

## 1. Introduction

Ovarian clear cell carcinoma (OCCC) is a distinct histotype of epithelial ovarian cancer (EOC), known for its unique molecular features, relative resistance to conventional therapies, and poorer clinical outcomes compared to other ovarian cancer subtypes [1,2,3]. Clear cell cancer of the ovary derives its name from the glycogen-rich cytoplasm which appears clear after formalin fixation [4,5]. OCCC represents 10–15% of all EOC cases in North America, but the incidence in Japan is in the order of 25% [6,7,8,9]. There is good evidence suggesting the endometrium as the OCCC cell of origin. Endometrial tissue undergoes retrograde transport during menses, and deposits on the surface of the ovary and other peritoneal organs [10,11,12]. Elegant research from several labs has demonstrated that endometriotic tissue in the peritoneal cavity can be contiguous with precancerous OCCC lesions, strongly suggesting that endometrial tissue is the source of OCCC [13,14,15,16,17]. When OCCC is found and surgically removed at Stage 1A, the 5 yr disease-free survival rate is very high at ~90% [18,19,20]. However, even with surgical debulking, late-stage OCCC is very difficult to treat, with a low response rate of 20–50% [21,22,23,24,25,26] to the gold-standard taxol and carboplatin-based chemotherapeutics [27,28,29,30,31]. Therefore, there is a significant need to identify alternative therapies that take advantage of the histotype-specific phenotype of epithelial ovarian cancers, including unique gene mutation status, homologous recombination deficiency, and the use of targeted agents such as bevacizumab and durvalumab for OCCC [32,33,34,35,36].

It is well established that EOCs do not spread via a hematogenous route and instead spread throughout the peritoneal cavity. A key mechanism of metastasis is the formation of multicellular clumps of tumor cells, termed spheroids, shed from the primary tumor [37,38,39,40,41]. Spheroids are resistant to anoikis-induced cell death and transit the peritoneal cavity through peritoneal fluid or the malignant ascites, if present. This allows spheroids to reattach to peritoneal surfaces, resulting in the growth of secondary tumors [42,43,44,45]. Hence, understanding the biology of tumor-derived spheroids is important to our understanding of the metastatic process. Moreover, spheroids represent an avascular 3D structure, distinct from 2D monolayer cultures, with a distinct metabolic and transcriptomic signature that allows survival in the hypoxic environment of the ascites fluid [46,47]. Therefore, we used ultralow-attachment (ULA) tissue culture plates to model spheroids in vitro, which allows human OCCC cell lines to autonomously form multicellular aggregates that recapitulate patient spheroid morphology. The spheroids can be transferred to adherent cell culture vessels which allow the spheroids to reattach and form a new monolayer of cells over time [48,49,50,51,52]. This in vitro model system is tractable and allows investigators to probe for vulnerabilities that can be therapeutically targeted to kill OCCC spheroid cells.

Despite its clinical significance, the biological mechanisms driving OCCC progression, particularly its ability to disseminate and metastasize within the peritoneal cavity, remain poorly understood. As described above, a defining feature of epithelial ovarian cancer, including OCCC, is the capacity of tumor cells to shed from the primary tumor and enter the peritoneal cavity, where they can form spheroids [46,47,53,54]. These three-dimensional cell clusters are resistant to anoikis and therapeutic intervention, contributing to metastatic spread and treatment refractoriness [42,53,54,55]. Notably, spheroids can adhere to the mesothelial lining of abdominal organs, where they establish metastatic lesions [55,56,57,58,59]. While spheroid formation plays a central role in the progression of ovarian cancer, the molecular mechanisms that govern OCCC spheroid survival and peritoneal dissemination remain largely undefined, particularly with respect to cell cycle checkpoint control and therapeutic resistance.

To address this gap in knowledge, we used OCCC cell lines to explore transcriptional and proliferative responses to spheroid culture conditions. We hypothesized that this divergence in how these cells avoid anoikis provides a unique opportunity to dissect the molecular pathways that regulate spheroid cell viability. Using integrative transcriptomic profiling through RNA sequencing (RNA-Seq), we characterized the global transcriptional landscape of each cell line under monolayer (ML) and spheroid (SPH) conditions.

Our analyses provide evidence that critical regulators of the cell cycle and mitotic progression are differentially expressed between proliferative and dormant spheroid cells. In addition to these intrinsic molecular mechanisms, we investigated potential therapeutic vulnerabilities in OCCC spheroids by targeting cell cycle checkpoint components using targeted inhibitors (Wee1 inhibitor, AZD1775). By directly comparing proliferative and dormant spheroid phenotypes, this study advances our understanding of OCCC biology within a physiologically relevant three-dimensional context. Our results provide a foundation for the development of novel therapeutic strategies aimed at disrupting spheroid-mediated metastasis and treatment resistance in OCCC.

## 2. Materials and Methods

### 2.1. Cell Culture and Reagents

Our sources of the 105C, KOC-7c, SMOV2, OVMANA, TU-OC-1, TOV-21G, and ES2 OCCC cell lines have been previously described [60]. The RMG-II and RMG-V cell lines were purchased from the Japanese Collection of Research Bioresources Cell Bank. The RMG-I and JHOC-5 cell lines were a gift from Dr. Hiroaki Itamochi, Iwate Medical University, Japan. Dr. Viji Shridhar (Mayo Clinic College of Medicine, Rochester, MN, USA) generously provided the OV207 cell line. Cells were maintained under conditions as previously described [60]. The JHOC-5 (LT66) cell line was generated in our lab by maintaining the JHOC-5 cell line for 66 consecutive days in a spheroid culture without intermittent trypsinization. The resultant cell line exhibits enhanced spheroid cell viability and growth. Briefly, cell lines were cultured in Dulbecco’s Modified Eagle Medium/Ham’s F12 (Invitrogen, Waltham, MA, USA, catalogue #11330057) supplemented with 10% FBS (Wisent, catalogue #098-150-CL). Cell lines were maintained as monolayer cultures grown at 37 °C with a 5% CO_2_. Once cells reached 80–90% confluency, media were aspirated. Then, cells were washed with Phosphate-Buffered Saline (PBS) (Wisent, Saint-Jean-Baptiste, QC, Canada, catalogue #311-425-CL), treated with trypsin/EDTA to induce detachment, and replated at 1:3–1:5 dilution. To drive spheroid formation, cells were seeded to ultralow attachment (ULA) plates (Corning, Corning, NY, USA, catalogue #3471 (6-well), 3473 (24-well), and 3474 (96-well)). Cell lines were authenticated using short tandem repeat (STR) analysis performed at the Centre for Applied Genomics (TCAG) in Toronto, Ontario, Canada and cross-referenced with STR fingerprints in the CLASTR 1.4.4 database (https://web.expasy.org/cellosaurus-str-search/, accessed on 8 March 2021).

### 2.2. Proliferation Assay

1 × 10^5^ cells were seeded in biological triplicates in 24-well ULA plates (Corning, catalogue #3473) in 1 mL DMEM:F12, supplemented with 10% FBS. On day 3, 1 mL of fresh medium was added to each well. To quantify cell number, the entire contents of each well were collected and centrifuged at 1500 rpm for 3 min. The resulting pellet was washed in PBS, resuspended in 50 µL of 0.25% trypsin (Wisent), and incubated at 37 °C for 15 min to dissociate cell clusters. Trypsin was neutralized by the addition of 100 µL FBS (Wisent, catalogue #098-150-CL). Cells were then counted by trypan blue exclusion using a TC20 Automated Cell Counter (Bio-Rad, Hercules, CA, USA).

### 2.3. Immunoblotting

For adherent monolayer cell cultures, whole-cell lysates were generated by gently scraping the cells into radioimmunoprecipitation assay (RIPA) buffer (50 mM Tris HCl, 150 mM NaCl, 1.0% (*v*/*v*) IGEPAL CA-630, 0.5% (*w*/*v*) Sodium Deoxycholate, 1.0 mM EDTA, 0.1% (*w*/*v*) SDS and 0.01% (*w*/*v*) sodium azide at a pH of 7.4) supplemented with 10% Sodium Deoxycholate, 225 mM sodium pyrophosphate, 0.5 M Sodium Fluoride, 200 mM Sodium Orthovanadate, 10×protease inhibitor cocktail, 200 mM PMSF, and 250 mM B-Glycerophosphate. The resulting mixtures were vortexed and kept on ice for 30 min with an additional vortexing step at 15 min. The lysates were then centrifuged at 14,000× *g* for 30 min at 4 °C. Supernatant was isolated, and protein concentration was quantified using the Bradford assay with Protein Dye Reagent (Bio-Rad, catalogue #500-0006).

For spheroid-derived protein lysates, cells were harvested from ULA vessels and centrifuged at 2400× *g* for three minutes. The supernatant was discarded and cell pellets were rinsed with ice-cold PBS after which they were lysed in a RIPA buffer on ice for 30 min; the remaining steps were the same as those for cells from monolayer cultures.

For electrophoretic separation, 20–30 µg of protein was loaded onto Bolt 4–12% Bis-Tris Plus polyacrylamide gels (Invitrogen, catalogue #NW04122BOX) and the reaction was run according to the manufacturer’s instructions. Protein transfer to PVDF (polyvinylidene difluoride) membranes was performed using the Bio-Rad “Trans-Blot Turbo” system. The blocking and washing of the PVDF membranes were conducted in Tris-buffered saline with Tween 20 (TBST; composed of 20 mM Tris, 150 mM NaCl, and 0.1% Tween 20) and blocked with either 5% skimmed milk or 5% bovine serum albumin (BSA), depending on the requirements of the primary antibodies used.

Primary antibodies were diluted according to the manufacturer’s recommendations, incubated with the membranes overnight at 4 °C, and then washed with three sequential washes of 10 min each with TBST. Membranes were then probed with either mouse or rabbit secondary antibodies for one hour at room temperature. Following secondary antibody incubation, membranes underwent three sequential washes of 10 min each with TBST. Detection was achieved by applying Immobilon Classico Western HRP Substrate (Millipore, Burlington, MA, US, catalogue #WBLUC0500) or Immobilon Forte Western HRP Substrate (Millipore, catalogue #WBLUF0500) for five min.

Finally, Western blot images were acquired, and the signal intensities were quantified using the Bio-Rad ChemiDoc imaging system.

### 2.4. Determining EC50 of AZD1775 for Monolayer (ML) and Spheroid (SPH) Cultured OCCC Cell Lines

The optimal starting cell number for each cell line and condition was determined empirically by plating the cells at different densities and determining which starting density resulted in a number of cells on day 5 that was within the dynamic range for the alamarBlue assay. Cells were then seeded in biological triplicates in a 96-well format at this optimal starting concentration. Ultralow-attachment plates (Corning, catalogue #3474) and regular tissue culture plastic (Sarstedt, Nümbrecht, Germany, catalogue #83.3920) were used for the suspension and monolayer, respectively. Then, 72 h after initial plating, cells were treated with AZD1775 (Cayman Chemical Company, Ann Arbor, MI, USA, catalogue #21266) at the concentrations listed (starting with a maximum concentration and serial dilutions across all wells).

For each drug concentration, the mean and standard deviation of the alamarBlue response signal across the three replicates were calculated. To enable curve fitting, the mean response values for each cell line and experiment were then normalized to a 0–100% scale. This normalization was performed by setting the minimum mean response within each dataset to 0% and the maximum mean response to 100%, effectively scaling all other mean response values proportionally within this range. The normalized mean responses, corresponding to each drug concentration, were then used to generate an EC50 curve using four-parameter logistic (4PL) regression. Non-linear least squares optimization, using the least squares function from the SciPy library, was employed to fit the 4PL model by minimizing the sum of the squared residuals between the observed normalized mean responses and the responses predicted by the 4PL model. EC50 values were determined directly from the fitted curve parameters. The visualization of fitted curves and normalized data points, including error bars representing standard deviation, was performed using matplotlib in Python (3.11).

### 2.5. siRNA-Mediated CDK1 Knockdown

The immunoblotting of CDK1 revealed two bands close to one another at the approximate weight of CDK1 (~33 kDa) in some samples. siRNA-mediated knockdown of *CDK1* (Gene ID: 983) was used to identify the CDK1-specific band. For the knockdown experiments, we used KOC-7c and JHOC-5 (LT66) cell lines grown in a monolayer, as well as KOC-7c grown as both a monolayer and a spheroid, as these lines showed strong CDK1 double banding when cultured as spheroids.

Cells were seeded at 2 × 10^5^ cells/well in 6-well vessels and treated with siRNA using DharmaFECT 1 transfection reagent (Dharmacon, Lafayette, CO, USA, cat. #T-2001-03) and ON-TARGETplus siRNA, targeting CDK1 (Horizon Discoveries, Cambridge, UK, catalogue #L-003224-00-0005), according to standard instructions. Three days post-treatment, 2 of the wells were harvested for Western blotting. Then, 4 of the remaining wells were combined and split between 3 wells of a 6-well ULA and a standard 10cm tissue culture dish (1 × 10^6^ cells). Three days after this replating (7 days post initial siRNA treatment) cells were harvested for Western blot. Western blot was performed on both sets of harvested extracts as described above.

### 2.6. RNA-Seq

#### 2.6.1. Sample Isolation

To obtain RNA from cell lines grown as a monolayer or in suspension, the 105C and KOC-7c cell lines were grown for 3 days on either tissue culture plastic or ULA plates. On day 3, RNA was extracted and purified using RNEasy Kit (Qiagen, Hilden, Germany, cat. 74106). Concentration and purity were measured using the NanoDrop One Microvolume UV-Vis spectrophotometer (Thermo Scientific, Waltham, MA, USA).

#### 2.6.2. Library Preparation

Library preparation and sequencing were performed at London Regional Genomics Centre at Robarts Research Institute, Western University. The quality of the isolated RNA was assessed using the Agilent 2100 Bioanalyzer (Agilent, Santa Clara, CA, USA). We used the VAHTS Total RNA-Seq (H/M/R) Library Prep Kit for Illumina (PN NR603) from Vazyme Biotech Co. Ltd. (Vazyme, Nanjing, China) to create indexed libraries which were sequenced using the Illumina NextSeq platform (150-bp; single-end reads). Raw data (fastq files) were generated and used for downstream mapping and analysis.

#### 2.6.3. Initial Processing

Initial processing was performed on the Galaxy platform. Raw sequences were confirmed to be of high-quality by analyzing per base sequence quality, GC content, N content, overrepresented sequences and adapter content using FastQC. Reads were mapped to the human genome (hg38) using HISAT2 using default settings and read counts were annotated to genomic features using featureCounts with default settings and built-in gene annotation files. Differential analysis was performed using limma, where genes without more than 0.5 Counts Per Million in at least 2 samples are insignificant and filtered out. TMM was the method used to normalize library sizes. eBayes was used with robust settings.

#### 2.6.4. Downstream Analysis

GSEA analysis: Gene set enrichment analysis (GSEA) was performed using the GSEA software (v4.4.0) from the Broad Institute of MIT and Harvard, following the method described by Subramanian et al. (2005) [61]. To obtain data amenable to GSEA analysis, we used limma contrast to compute the difference of differences, specifically [(KOC-7c SPH–KOC-7c ML)–(105C SPH–105C ML)]. This approach allowed us to determine the genes that exhibited a differential behavior between KOC-7c and 105C across the two growth conditions. “GSEAPreranked” was used as described by Reimand et al. [62]. Briefly, the ranked gene list was obtained by taking the −log10 (*p*-value) from limma output and multiplying it by the sign of the log-transformed fold change.

GSEA heatmap generation: gene sets enriched in GSEA KOC-7c (SPH/ML)/105C (SPH/ML) were obtained from GSEA output.

Gene ontology (GO) analysis: To perform GO gene set enrichment analysis using the GO Biological Processes and GO Cellular Components datasets, we first identified genes that were differentially expressed between 105C (spheroid/monolayer) and KOC-7c (spheroid/monolayer) using an adjusted *p* value < 0.05 and separated them into lists of genes whose expression was significantly higher in 105C (spheroid/monolayer) vs. KOC-7c (spheroid/monolayer) as well as the opposite, higher in KOC-7c (spheroid/monolayer) vs. 105C (spheroid/monolayer). We then used the Enrichr platform [62,63,64,65] to perform the analysis using default settings.

Principle component analysis (PCA): a PCA plot was generated using the PCA module from the sklearn python library.

General analysis: Unless otherwise indicated, bar plots, scatter plots, and heatmaps depicting expression levels or fold changes were generated from raw or pre-processed data (e.g., fold changes generated by limma) using custom python scripts and common libraries—numpy, seaborn, matplotlib, Pandas. Scripts available upon request.

### 2.7. Spheroid Reattachment Assay

OCCC cell lines were seeded in 24-well ULA culture plates at 2.5 × 10^4^ cells/well for KOC-7c and 1 × 10^5^ cells/well for 105C. Cells were treated 3 days post-seeding, in biological triplicates, to allow spheroids to form. After 3 days of treatment, spheroid cells were transferred (along with media), well for well, into 24-well adherent culture plates, and then supplemented with additional DMEM/F12 + 10% FBS media. For KOC-7c cells, spheroid cells were allowed to attach to cell culture substrata overnight. This was allowed for over 48 h for 105C cells. Once attached, cell viability was quantified using alamarBlue cell viability reagent, and fluorescence was measured at 590 nm using the Agilent BioTek Synergy H1 (Agilent).

### 2.8. AZD1775 Time Course

For monolayer time course experiments, KOC-7c and 105C cells were seeded in 10 cm adherent culture plates (Sarstedt, Newton, NC, USA) at densities that yielded a ~90% confluent plate at the time of protein collection. For spheroid time course experiments, KOC-7c cells were seeded at 2 × 10^5^ cells/well and 105C cells were seeded at 1 × 10^6^ cells/well in 6-well ULA culture plates. For both culture conditions, cells were treated 3 days post-seeding with AZD1775 and whole-cell extracts were collected at every time point for Western blot analysis of total CDK1, p-CDK1, γH2AX, pH3, cleaved Caspase-3, and cleaved PARP.

### 2.9. Antibodies

The primary and secondary antibodies used in this study to assess steady-state levels of a variety of proteins by western blotting is listed in Table 1 (below). We also provide the commercial supplier of each used in our experiments.

## 3. Results

### 3.1. Distinct Proliferative and Transcriptional Responses of Ovarian Clear Cell Carcinoma Cell Lines to Suspension Culture

**Figure 1:**
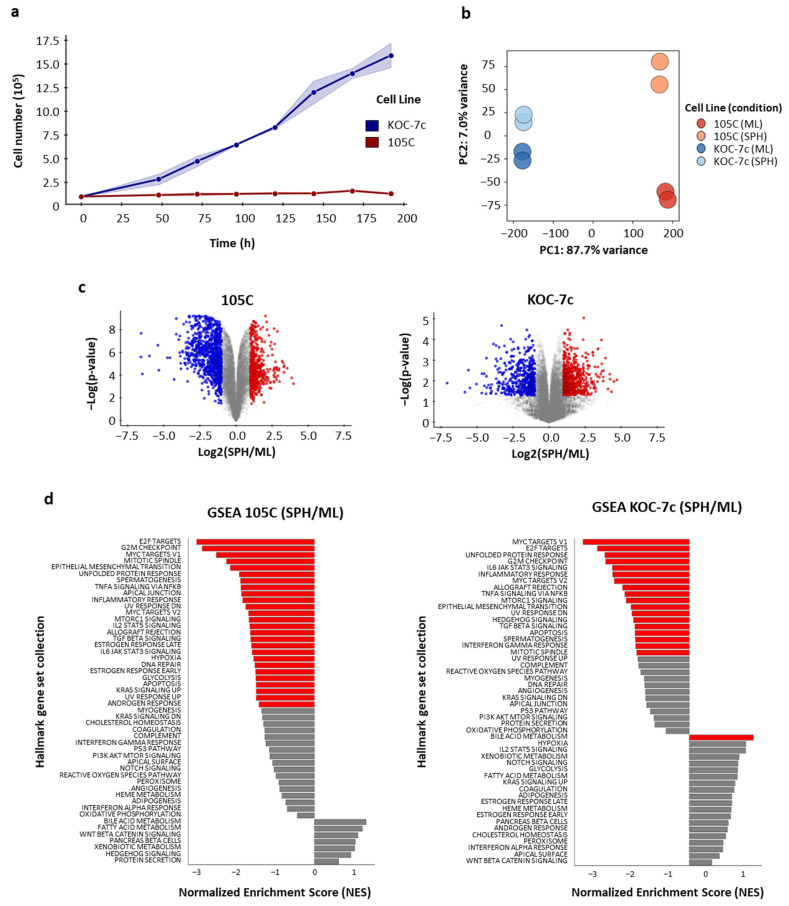
RNA-Seq analysis of ovarian clear cell carcinoma cell lines 105C and KOC-7c, comparing the monolayer to spheroid transcriptome. (**a**) Change in cell number of KOC-7c and 105C cell lines maintained over time in suspension culture using ultralow-attachment plates. The doubling time for the KOC-7c spheroid cells is 57 h, whereas the 105C cell line spheroid cell number does not change over time after 5 days in suspension culture. (**b**) PCA plot on RNA-Seq data showing the distinct grouping of 105C and KOC-7c cell lines and further separation between cells grown as monolayer (ML) compared to those maintained in suspension as spheroids (SPH) within each group, indicating that the largest differences between the samples are primarily due to cell types with further differences seen as a consequence of culture conditions especially for the 105C line. (**c**) Volcano plots of RNA-Seq data for both 105C and KOC-7c cell lines. Differentially expressed genes (DEGs) in 105C and KOC-7c cell lines observed when comparing expression between monolayer and spheroid within each line with the 105C cell line showing a much larger number of genes exhibiting significant changes in expression while the transcriptome of the KOC-7c line does not change significantly between monolayer and spheroid culture (red (upregulated); blue (downregulated): *p* < 0.05 and absolute (fold change) > 2). (**d**) GSEA analyses of differentially expressed genes in 105C and KOC-7c lines reveals pathways downregulated when cells are grown in suspension as 3D spheroids (red bars: FDR adjusted *p*-value < 0.05).

We have previously shown that human OCCC cell lines respond differently to suspension cultures that facilitate the autonomous formation of 3D clusters or spheroids [60]. Some OCCC cell lines can proliferate (KOC-7c) in spheroid cultures while others enter a dormant state (105C). Moreover, the 105C and KOC-7c cell lines carry a gene mutational profile typical of OCCC, with mutated *PTEN* and *PIK3CA* genes resulting in hyperactivated *AKT* signaling along with mutated *ARID1A* and wildtype TP53 [60,66,67,68,69]. We cultured the 105C and KOC-7c cell lines on ultralow-attachment (ULA) plates, which prevented cells from attaching to the substratum, in DMEM/10% FBS, for 200 h (Figure 1a). KOC-7c cells clearly proliferated over time as 3D spheroids while the 105C cell line remained relatively static during the same period. These results indicate a marked difference in the proliferative capacity of the two cell lines in spheroid form.

To investigate the cellular programs underlying the observed spheroid phenotypic differences between these two OCCC cell lines, RNA-Seq was performed in duplicate, interrogating these lines under two culture conditions: monolayer (ML) and spheroid (SPH) (Figure 1b, Table S1). Differential gene expression analyses identified a total of 5168 differentially expressed genes (DEGs) in the 105C cell line and 1557 DEGs in the KOC-7c cell line when comparing spheroid to monolayer conditions.

Principal component analysis (PCA) was conducted to assess the overall transcriptional differences between the 105C and KOC-7c in monolayer and spheroid culture conditions (Figure 1c). Not surprisingly, the first principal component (PC1) accounted for 87.7% of the total variance and primarily separated the samples based on cell line identity, distinguishing 105C from KOC-7c. The second principal component (PC2) explained 7.0% of the variance and segregated the samples according to their culture conditions (ML vs. SPH). Notably, KOC-7c samples exhibited tighter clustering irrespective of culture conditions, whereas 105C samples demonstrated distinctly broader separation between ML and SPH conditions, indicating greater variability in gene expression profiles under 2D versus 3D cultures.

Next, we applied Gene Set Enrichment Analysis (GSEA) to the RNA-Seq data to reveal biological pathways significantly impacted by culture conditions (Figure 1d). In the 105C cell line, 26 pathways were significantly enriched, with all pathways showing negative normalized enrichment scores (NESs) indicating downregulation in 3D SPH compared to proliferating ML. The KOC-7c cell line exhibited 19 significantly enriched pathways, with 18 pathways demonstrating negative NES values (downregulation) and 1 pathway (Bile Acid Metabolism) showing upregulation, in SPH relative to ML.

### 3.2. Comparative Pathway and Gene Expression Dynamics in Ovarian Clear Cell Carcinoma Cell Lines Under Spheroid and Monolayer Growth Conditions

**Figure 2:**
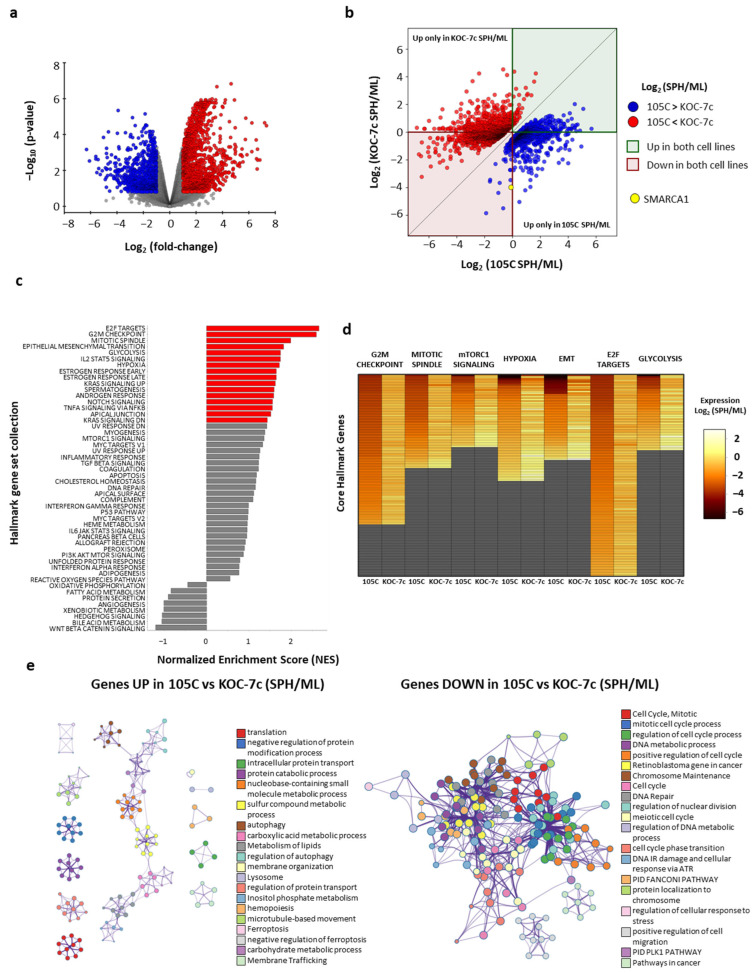
Pathways are differentially regulated in response to spheroid cultures between 105C and KOC-7c cell lines. (**a**) Volcano plot using genes that are differentially expressed when comparing response of 105C to KOC-7c maintained in spheroid cultures. Red markers indicate genes that are significantly increased in KOC-7c cells in response to spheroid cultures relative to 105C cell line response to spheroid culture, while blue markers signify genes whose expression is lower in response to KOC-7c spheroid culture relative to 105C cell line in spheroid cultures. (**b**) Comparing change in gene expression between 105C and KOC-7c under monolayer or spheroid culture conditions, which are identified as being significantly different between KOC-7c (SPH/ML) vs. 105C (SPH/ML). Each marker represents fold change of gene (suspension/monolayer) for both 105C and KOC-7c cell lines. Red dots signify genes significantly upregulated in KOC-7c cells relative to 105C cells based on ratio of spheroid to monolayer data. Blue dots indicate genes significantly downregulated in KOC-7c cells relative to 105C based on ratio of spheroid to monolayer. The red quadrant contains genes which are increased in both 105C (SPH/ML) and KOC-7c (SPH/ML), while the green quadrant contains genes that are elevated in both 105C (SPH/ML) and KOC-7c (SPH/ML). Genes in red and green quadrants have same direction of change with regard to expression (SPH/ML); however, magnitude of change in gene expression between 105C and KOC-7c cell lines is sufficient to cause genes to be seen as significantly down- or upregulated in KOC-7c (SPH/ML) relative to 105C (SPH/ML). White quadrants contain genes which are regulated in opposite directions across each cell line, meaning that distribution of DEGs is predominantly cell-specific. The yellow point highlights *SMARCA*, a gene that is downregulated in both 105C and KOC-7c (SPH/ML); however, due to magnitude difference, it is still seen as reduced in KOC-7c vs. 105C; (**c**) GSEA analysis using difference in fold change between two cell lines for each gene (i.e., (KOC-7cSPH/KOC-7cML)/(105CSPH/105CML)] reveals multiple pathways that are differentially regulated when comparing response of 105C and KOC-7c cell lines to 3D spheroid formation (red bars: FDR adjusted *p*-value < 0.05). (**d**) Heatmap of fold change (suspension/monolayer) of core GSEA genes composing pathways that were identified as significantly enriched in GSEA analysis reveals that 105C cell line downregulates expression of core genes when cultured as spheroids (relative to monolayer) while KOC-7c cell line generally maintains consistent expression of these genes between monolayer and spheroid culture; (**e**) Metascape analyses describing genes upregulated (on left) in 105C vs. KOC-7c form in contrast, distinct functional clusters which are not overlapping and enriched in pathways such as translation, autophagy, metabolic processes, and ferroptosis regulation indicating shift towards stress adaptation and metabolic reprogramming. Right side Metascape Network analysis of genes downregulated in 105C vs. KOC-7c (SPH/ML) reveal highly interconnected network primarily enriched in pathways related to cell cycle regulation, DNA repair, and chromosome maintenance suggesting reduction in proliferative and genome maintenance processes in 105C cell line relative to spheroid proliferative KOC-7c line. Nodes represent biological terms and edges, or interconnecting lines define term overlap based on shared gene memberships. Colors correspond to pathway annotations.

To identify pathways differentially regulated in response to spheroid versus monolayer growth conditions between the two ovarian clear cell carcinoma cell lines, comparative fold change analysis was conducted that identified genes differentially regulated between the lines in response to being grown in suspension (Figure 2a and Table S2). The volcano plot illustrates that a distinct transcriptional response exists between lines when cultured in suspension, with 2911 genes being differentially regulated (1741 up and 1170 down in KOC-7c (SPH/ML) compared to 105C (SPH/ML).

To further elucidate how the expression of these genes changes in 105C and KOC-7c cells, for each differentially expressed gene, we compared its fold change (SPH/ML) within each line (Figure 2b and Table S2). Out of the 2911 genes showing a differential response between lines, 1550 genes (53% of DEGs) are regulated in opposite directions within the lines (upregulated in one and downregulated in the other). Interestingly, 1361 genes (47% of DEGs) are regulated in the same direction within both lines (464 genes downregulated and 901 genes upregulated in SPH versus ML). However, the extent of the expression change for many of these genes still differs enough between the two lines and culture conditions for them to be classified as differentially regulated. The dichotomy of transcriptome changes in spheroid cells in 105C versus KOC-7c spheroid cells is depicted in each quadrant of Figure 2b. The number of genes elevated in each cell line as spheroids compared to the proliferating monolayer cells is similar (1317 genes for KOC-7c and 1166 for 105C). When analyzing genes whose expression is elevated in spheroid cells of both lines (green quadrant), we see that 414 genes are upregulated in 105C spheroid cells relative to KOC-7c spheroids, whereas in KOC-7c spheroid cells, only 50 genes are significantly upregulated relative to 105C spheroid cells. In the red quadrant, genes are downregulated in spheroid cells of both lines relative to their monolayer counterparts. However, 841 of these genes are downregulated to a greater magnitude in the 105C spheroid cells compared to the KOC-7c spheroids. Only 61 genes are downregulated more extensively in the KOC-7c spheroid cells relative to the 105C spheroids. For example, even though *SMARCA1* (Gene ID: 6594), a SWI/SNF complex member responsible for chromatin remodeling [70], is reduced in both 105C (log2 (SPH/ML) = −0.11) and KOC-7c (log2 (SPH/ML) = −3.98) spheroid cells it is still identified as being reduced in KOC-7c as [KOC-7c (SPH/ML)/105C (SPH/ML)] = 0.07, and exceeds the threshold (*p* < 0.05). These findings highlight both the shared and unique transcriptional responses of the 105C and KOC-7c cell lines to spheroid formation.

To identify cancer hallmark pathways differentially enriched in KOC-7c compared to 105C under spheroid growth conditions relative to monolayer conditions, we performed gene set enrichment analysis (GSEA) on all datasets (Figure 2c). Key pathways identified as statistically significant and enriched in KOC-7c spheroid cells included the G2/M checkpoint, E2F targets, epithelial-mesenchymal transition (EMT), and glycolysis. In contrast, pathways associated with inflammation and apoptosis were downregulated in KOC-7c when cultured as spheroids compared to 105C spheroids relative to monolayer cells.

Further examination of the gene-level expression of the core GSEA hallmark genes revealed that the 105C cell line generally downregulated these genes in a much more pronounced manner under spheroid conditions compared to monolayers. This is indicated by the heatmaps showing gene expression changes for each cell line when comparing spheroid to monolayer cells. Conversely, the KOC-7c cell line maintained or increased the expression of these genes when grown as spheroids relative to monolayers as depicted by the heatmap (Figure 2d). This pattern indicates the existence of distinct transcriptional responses between the two cell lines in response to spheroid formation. The upregulation of key cell cycle and metabolic pathways in KOC-7c suggests that this cell line may rely more heavily on these processes when cultured in suspension compared to 105C, further reinforcing the hypothesis that KOC-7c demonstrates a more viable and proliferation-specific phenotype under spheroid conditions.

To gain further insight into the functional connections between the differentially regulated genes, we generated a network plot for both up- and downregulated genes (105C (SPH/ML) and KOC-7c (SPH/ML)) using Metascape Network analysis (Figure 2e). This revealed strikingly different profiles for genes that were up- and downregulated, with the downregulated gene set exhibiting significant enrichment for cell cycle-related pathways, DNA repair, and chromosome maintenance, forming a single highly interconnected network in both lines (Figure 2e, right). In contrast, the upregulated genes mapped to multiple discrete clusters, including pathways in translation, autophagy, protein catabolism, and intracellular transport (Figure 2e, left). These findings suggest that 105C spheroid cells exhibit lower expression of core cell cycle and DNA maintenance genes (consistent with the observed difference in spheroid cell proliferation between these cell lines), while upregulating a range of processes linked to protein homeostasis and metabolic pathways to enhance cell survival as 3D spheroids.

**Figure 3:**
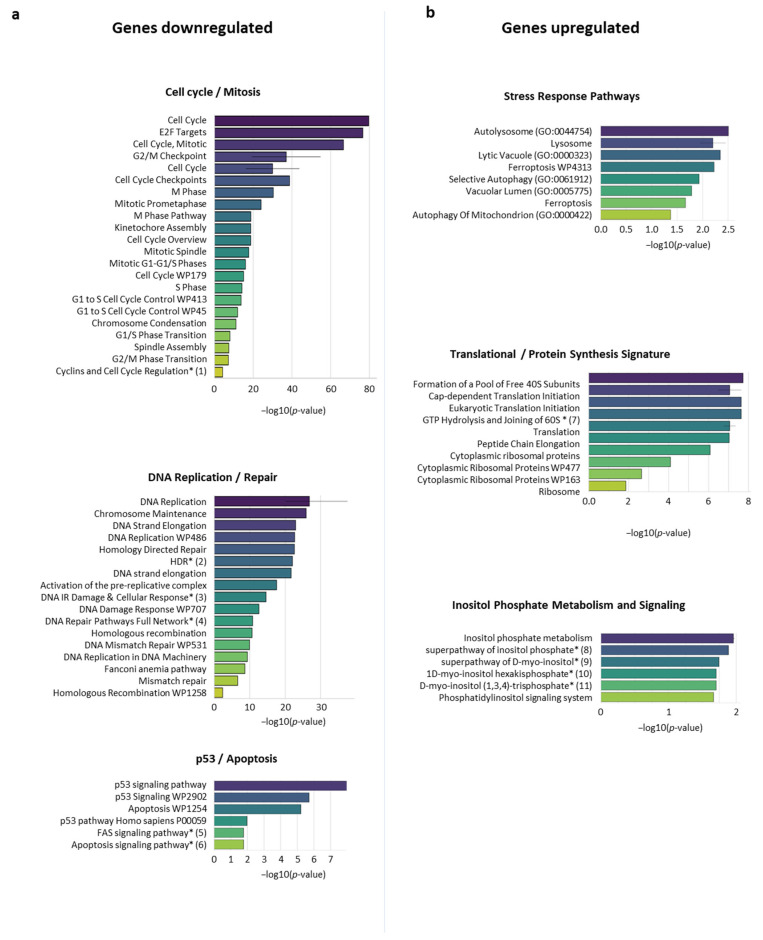
Enrichr analysis identified pathways found to be differentially expressed in 105C SPH/ML vs. KOC-7c SPH/ML. To identify pathways that are potentially differentially regulated between 105C and KOC-7c cell lines when maintained in suspension, we performed gene set enrichment using Enrichr for genes downregulated in response to suspension culture in 105C vs. KOC-7c cells as well as those which are upregulated. (**a**) Genes which are downregulated in 105C (SPH/ML) relative to KOC-7c (SPH/ML) reveal enrichment for terms associated with cell cycle/mitosis DNA replication/repair and p53/apoptosis. (**b**) Upregulated genes instead show an enrichment relating to translation stress response as well as energy homeostasis (Inositol Phosphate Metabolism and Signaling). * indicates terms that are truncated in this Figure for brevity.

To identify pathways which are differentially controlled and perhaps contribute to the formation and viability of spheroid cells, we employed the Enrichr search engine to compare our RNA-Seq data to the Enrichr annotated gene set. This approach facilitates the analyses of transcriptomic data to identify biochemical pathways relevant to the model system under investigation. This approach revealed multiple pathways whose core gene complement was differentially expressed between the 105C (SPH/ML) and KOC-7c (SPH/ML) cell populations (Figure 3). These analyses further confirmed the data obtained with GSEA analysis and functional mapping (Metascape Network), given that several cell cycle/mitotic pathways and core DNA replication/repair signatures were significantly downregulated in 105C (SPH/ML) relative to the KOC-7c cell line. Interestingly, despite the initial expectation that apoptosis would be lower in the suspension-growing KOC-7c line, our analysis revealed the surprising downregulation of the apoptosis signature in 105C cells. When analyzing gene expression across conditions, we observed that 105C cells decrease the expression of all apoptosis-related genes in suspensions, including anti-apoptotic and context-dependent genes defined, as encoding apoptotic proteins whose regulation is dependent on the cell environment. This suggests that the observed downregulation of the apoptosis signature in 105C does not necessarily indicate reduced apoptosis compared to KOC-7c, but rather the general suppression of both pro- and anti-apoptotic factors in spheroid cells.

Genes upregulated in 105C (SPH/ML) vs. KOC-7C (SPH/ML) were enriched for stress response signatures (“Autophagy”, “Ferroptosis”, “Mitochondrion Disassembly”) as well as translational and protein synthesis pathways (e.g., “Cap-Dependent Translation Initiation”, “Peptide Chain Elongation”) (Figure 3b and Figure S1). In addition, inositol phosphate metabolism and signaling pathways (e.g., “Superpathway of D-myo-inositol”, “Phosphatidylinositol Signaling”) emerged as strongly enriched among genes that showed an increase in expression in the 105C spheroid cells compared to a monolayer cell population. Overall, these differences indicate that 105C spheroid cells exhibit a notable shift away from cell cycle progression and DNA repair processes toward stress adaptation and protein translation signatures contributing to 105C spheroid cell viability relative to KOC-7C spheroid cells which maintain a proliferative phenotype.

Truncated terms:

1. Cyclins and cell cycle regulation Homo sapiens h cell cycle pathway;
2. HDR through homologous recombination (HRR) or single-strand annealing (SSA);
3. DNA IR damage and cellular response via ATR WP4016;
4. DNA repair pathways full-network WP4946;
5. FAS signaling pathway Homo sapiens P00020;
6. Apoptosis signaling pathway Homo sapiens P00006;
7. GTP hydrolysis and joining of the 60S ribosomal subunit;
8. Superpathway of inositol phosphate compounds Homo sapiens PWY-6371;
9. Superpathway of D-myo-inositol (1,4,5)-trisphosphate metabolism Homo sapiens PWY-6358;
10. 1D-myo-inositol hexakisphosphate biosynthesis I (mammalian) Homo sapiens PWY-6362;
11. D-myo-inositol (1,3,4)-trisphosphate biosynthesis Homo sapiens PWY-6364.

### 3.3. Role of G2/M Checkpoint Regulation in Dormant and Proliferative OCCC Spheroids and Sensitivity to Wee1 Inhibition

**Figure 4:**
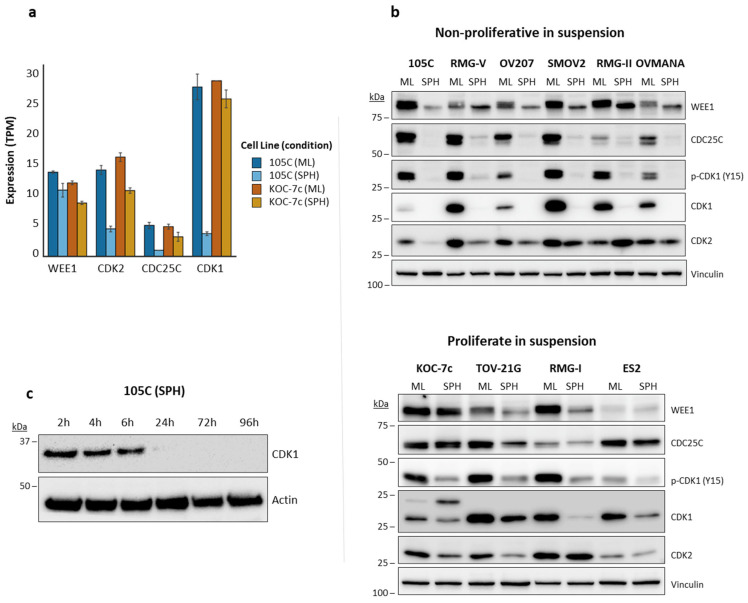
Expression of G2/M pathway proteins. (**a**) G2/M pathway gene transcript levels (Transcripts Per Million) on day 3 105C and KOC-7c monolayer or spheroid cells shows downregulation of CDK1 and CDC25C expression in 105C spheroid cells. Data are mean ± SEM of *n* = 2 independent RNA-seq experiments. (**b**) Western blot analysis of G2/M pathway protein levels in day 3 OCCC cell lines cultured as monolayer or spheroid cells. OCCC cell lines that do not proliferate as spheroids show consistent downregulation of G2/M pathway proteins when cultured as spheroids while expression is more likely to be maintained in cell lines that proliferate in spheroid culture. (**c**) Time course using whole-cell lysate was collected from 105C cells cultured in suspension at each indicated time point and incubated with antibody targeting CDK1. We used siRNA knockdown of CDK1 to show that higher molecular weight band that we observe is non-specific (Figure S2).

Our bioinformatic analyses clearly pointed to cell cycle regulation and the G2/M checkpoint as vulnerabilities in proliferating spheroid cells exemplified by the KOC-7c cell line. To determine if key G2/M cell cycle regulators are differentially expressed in 105C and KOC-7c spheroid cells, we surveyed the protein levels of Wee1, CDC25C, and CDK1 in ten OCCC cell lines, grown as monolayers or as spheroids to determine whether protein levels were consistent with transcript levels obtained from our RNA-Seq data (Figure 4a,b). Our previous work showed that some OCCC cell lines can form dormant spheroids (105C, RMG-V, OV207, SMOV2, RMG-II, OVMANA) [60]. These lines exhibited a consistent and strong downregulation of Wee1, CDC25C, the repressive phosphorylation of CDK1 at Tyr15 (p-CDK1), and total CDK1 protein levels in day 3 spheroid cells. OCCC cell lines with actively proliferating spheroids (KOC-7c, RMG-I, TOV-21G, and ES2; [60]) were more likely to maintain the expression of G2/M regulators in spheroid form compared to non-proliferating spheroids, as was most evident from CDC25C levels.

A decrease in p-CDK1 level was always seen when OCCC cells were cultured as spheroids compared to adherent cells. The downregulation of p-CDK1 is mainly facilitated by a decrease in total CDK1 protein in non-proliferating spheroid cells. A similar but much less intense trend showing the loss of CDK1 and p-CDK1 was also observed in proliferating spheroid cells, even though these cells continue to grow.

We also asked how quickly detectable CDK1 levels are lost in dormant, non-proliferative spheroid cells. We maintained the 105C cell line spheroids in suspension culture and collected whole-cell extracts from 2 to 96h. We observed that CDK1 produces a noticeably weaker signal at 6h and is undetectable by 24h (Figure 4c). Together, these results show that a decrease in G2/M pathway protein levels correlates with OCCC spheroid cell dormancy that supports our RNA-Seq results as applicable to spheroid-induced transcriptomic changes in a wide range of established OCCC cell lines.

**Figure 5:**
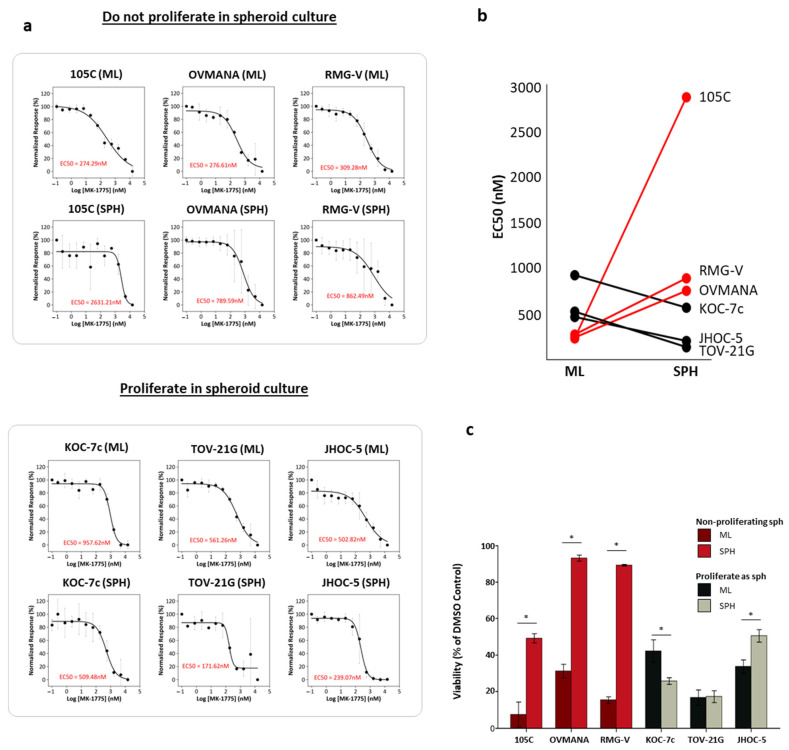
Wee1 inhibitor AZD1775 EC50 determinations on OCCC cell lines which behave differently in spheroid cultures. (**a**) Cells were cultured for three days either in monolayer or suspension followed by a 3-day treatment with AZD1775 at concentrations ranging from 1 nM to 15 µM (*n* = 3) and then alamarBlue was used to measure cell viability. Measurements were normalized to DMSO (100%) (0% = value at highest concentration). (**b**) EC50 values indicate that lines which do not proliferate (black lines and points) appear to be more sensitive as spheroids compared to their monolayer counter parts, while lines which do not proliferate as spheroids (red line and points) appear to become more resistant. See Supplementary Table S3 for actual EC50 values for each cell line and culture condition. (**c**) At maximum concentration of AZD1775, we tested (15 µM) cell lines which do not proliferate in suspension showed an increase in viability as spheroids when compared to their monolayer counterparts. Cells which do proliferate showed decreased viability or no change. JHOC-5 both does not proliferate in suspension during early time points but shows robust proliferation after day 10 (Supplementary Figure S3). Data are mean ± SEM of *n* = 3 independent experiments; * *p* < 0.05 versus corresponding monolayer (two-tailed paired *t*-test). Red line: ML(EC50) < SPH(EC50); Black line: ML(EC50) > SPH(EC50).

Given the dramatic differences in G2/M cell cycle regulator expression between proliferating and non-proliferating spheroid cells, components of the G2/M checkpoint could represent viable drug targets against spheroid cells. It is also well established that autonomously aggregated cancer cell spheroids form unique cell–cell contacts and communication, and spatially distinct areas of metabolism [53,56,71,72,73,74], different from 2D monolayer cell cultures. G2/M transition is primarily regulated by Wee1, CDC25A and CDC25B, as well as ATR and CHK1 [75,76,77]. Wee1 is the main regulator of the G2/M checkpoint and its phosphorylation of Tyr15 of CDK1 is responsible for checkpoint blockade [78,79,80]. The Wee1 inhibitor, Adavosertib or AZD1775, is currently in clinical trials for several different solid tumors [81,82,83,84] and is therefore an obvious therapeutic option to investigate efficacy on OCCC cell line spheroids. Given that a reduction in Wee1 activity results in mitotic entry prior to efficient DNA repair, leading to mitotic catastrophe and cell death [85,86,87,88,89], we hypothesized that using AZD1775 to target the enzymatic activity of Wee1 might effectively kill spheroid cells from both proliferative and dormant OCCC spheroids. To establish the optimal concentration of AZD1775 for use in downstream analysis, we established the EC50 for six cell lines (3 days of treatment) under both monolayer and spheroid culture conditions: three cell lines which proliferate as spheroids (i.e., KOC-7c, TOV-21G, JHOC-5) and three that do not proliferate (i.e., 105C, OVMANA, RMG-V) (Figure 5a,b, Supplementary Table S3). These data are displayed as a Normalized Response (%), where 0% indicates the maximum response observed in the cell line at the highest dose and 100% indicates no response. Notably, OCCC cell lines which proliferated in suspension showed an increase in sensitivity with EC50 values 1.5–3.2-fold lower when treated as spheroids relative to 2D cultures, while the lines which do not proliferate as spheroids exhibited higher EC50 values at 2.8- to 10.6-fold greater than monolayer cells (Figure 5b). We provide a graphical representation, showing the viability of each cell line under spheroid and monolayer conditions at the maximum dose of AZD1775 (Figure 5c). The non-proliferating spheroids (105C, OVMANA, RMG-V) have much higher viability than the proliferating spheroids (KOC-7c, TOV-21G, JHOC-5) when treated with the highest dose (15 µM).

**Figure 6:**
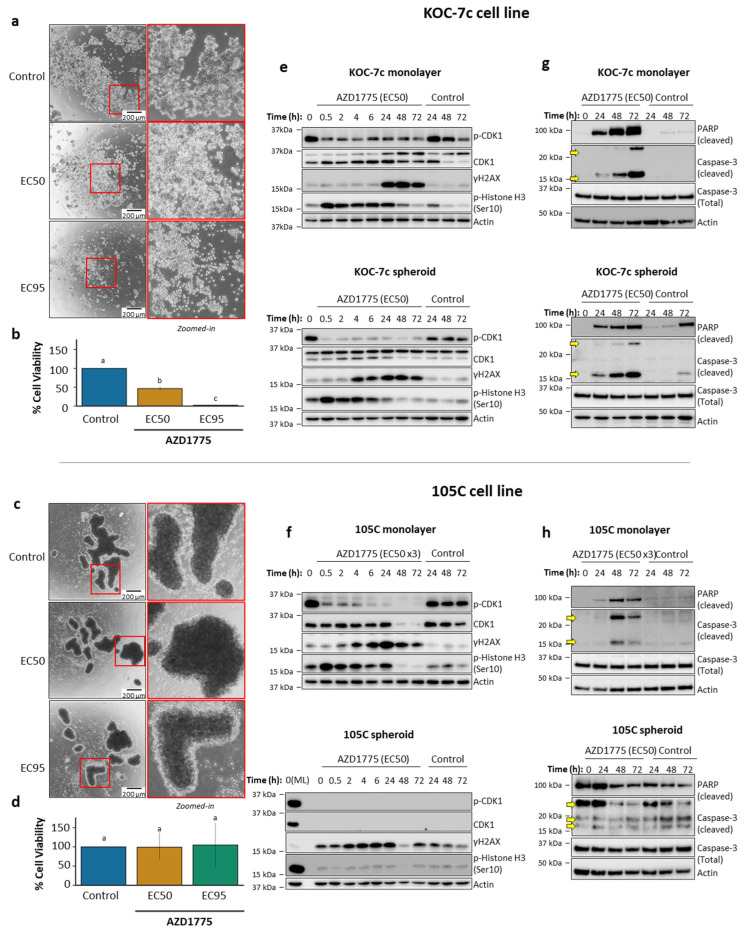
Effects of AZD1775 on mitotic entry and DNA damage in 105C and KOC-7c cells in monolayer and spheroid culture. (**a**,**c**) Representative images of KOC-7c and 105C spheroids treated with AZD1775. KOC-7c spheroids were seeded at 1 × 10^4^ cells/well in 24-well ultralow-attachment (ULA) plates allowed to form for 3 days and then treated for an additional 3 days. Similarly, 105C spheroids were seeded at 1.25 × 10^5^ cells/well in 24-well ULA plates also treated 3 days after seeding and imaged following 3 days of treatment. All images were taken immediately before reattachment. Red boxes indicate regions shown at higher magnification; (**b**,**d**) 105C and KOC-7c spheroid reattachment assay results after treatment with AZD1775. Cells were seeded at density of 1.25 × 10^5^ cells per well for 105C cell line and 1 × 10^4^ cells per well for KOC-7c cell line in 24-well ULA plates. Three days after seeding, cells were treated with their respective AZD1775 EC50 and EC95 concentrations for three days. 105C cell line EC50 was ~3 µM and EC95 was ~9 µM while for KOC-7c cell line EC50 was ~600 nM and EC95 was ~3 µM. Following three days of treatment cells were reattached into 24-well adherent culture plates. Cell viability after reattachment was determined using alamarBlue assay. 105C cells exhibited resistance to AZD1775 at both doses of drug. Error bars represent SEM. Conducted one-way ANOVA followed by Tukey’s multiple comparisons test. Different letters represent significantly different values (*p* < 0.05); (**e**,**f**) Cells were treated with their respective AZD1775 EC50 concentrations for 3 days (105C monolayer cells treated with triple their EC50). Western blot analysis performed for phospho-CDK1 total CDK1, γH2AX, and phospho-H3. Actin was used as loading control; (**g**,**h**) Adherent (monolayer) cells for both cell lines were seeded in 6-well adherent culture plates at densities that yielded ~90% confluency at time of lysate collection. Spheroid cells were seeded at 2 × 10^5^ cells/well for KOC-7c cells and 1 × 10^6^ cells/well for 105C cells in 6-well ULA culture. KOC-7c cells were treated with their AZD1775 EC50 concentration (ML: 950 nM; SPH: 600 nM). 105C adherent cells were treated at triple their AZD1775 EC50 concentration (750 nM) and 105C spheroid cells were treated at EC50 of KOC-7c spheroid cells. Cells were treated 3 days post-seeding and whole-cell protein lysates were collected at every time point and used for Western blot analysis of apoptosis markers. Different letters (a, b, c) represent significantly different values (*p* < 0.05). Yellow arrows denote cleaved Caspase-3 bands.

To further support the EC50 experiments, we performed spheroid reattachment assays as a rigorous measure of spheroid cell viability upon treatment with AZD1775 in the 105C and KOC-7c cell lines (Figure 6a–d). The assay utilizes the ability of spheroids to re-attach to the cell culture substratum after treatment. This allows us to model the formation of secondary lesions during epithelial ovarian cancer (EOC) metastasis. KOC-7c spheroids displayed a statistically significant, concentration-dependent decrease in cell viability when treated with 600 nM (EC50) and 3 µM (EC95) AZD1775, with cell viability completely ablated upon treatment with the EC95 concentration. In contrast, 105C spheroids displayed a marginal decrease in cell viability (not statistically significant) when treated with 3 µM (EC50) and 9 µM (EC95) AZD1775, showing that 105C spheroids are refractory to the “maximal” inhibitory concentration of a drug. The inhibition of Wee1 severely impacts the viability of proliferating spheroid cells, but is less efficacious in terms of non-proliferating OCCC spheroids.

To elucidate the mechanism of cytotoxicity, we treated KOC-7c and 105C cells at several timepoints and probed for CDK1, p-CDK1, phosphorylation of histone H3 at Ser10 (p-Histone H3 (Ser10)) and histone variant H2AX at Ser139 (γH2AX) (Figure 6e,f and Figure S4). KOC-7c and 105C cells were treated at their AZD1775 EC50 concentrations as both adherent and spheroid cells, and subsequent Western blot analysis was performed. We observed a clear diminution in p-CDK1 levels by 30 min after the addition of AZD1775 in both KOC-7c adherent and spheroid cells. The p-Histone H3 (Ser10) and γH2AX were used to describe the mitotic progression and DNA damage response, respectively [90,91]. Phospho-Histone H3 levels peaked 30 min after treatment and remained elevated until 24h as cells became progressively less proliferative due to the accumulation of DNA damage, seen through the elevation in γH2AX levels at later time points. Interestingly, neither p-Histone H3 nor γH2AX levels were elevated over the duration of treatment in 105C adherent cells treated at the EC50 (~250 nM) despite a substantial decrease in inhibitory p-CDK1 levels. At 3 × AZD1775 EC50 (~750 nM), an increase in these markers, similar to the response seen in KOC-7c cells, was evident. There was no detectable CDK1 (and consequently, p-CDK1) protein in 105C spheroids for the duration of AZD1775 treatment. As expected, p-Histone H3 and γH2AX levels stayed relatively consistent throughout the treatment period. These results support our observation that AZD1775 is most effective in proliferating spheroid cells as occurs in the KOC-7c cell line.

### 3.4. Effects of AZD1775 on Apoptosis in 105C and KOC-7c in ML and SPH

We also determined the effects of AZD1775 on apoptosis in KOC-7c and 105C monolayer and spheroid cells (Figure 6g,h). AZD1775 treatment clearly elevated both cleaved Caspase-3 and cleaved PARP levels relative to the vehicle in KOC-7c cells after just 24h of treatment. The timeframe for apoptosis marker expression aligned with that of γH2AX in KOC-7c cells in either monolayer or 3D spheroid cultures. The treatment of 105C monolayer cells at their EC50 concentration did not result in a detectable elevation of cleaved Caspase-3 and little elevation in cleaved PARP levels (Figure S5), while triple the EC50 concentration (750 nM) resulted in the elevation of both apoptosis markers by 48h of treatment. In support of previous results, AZD1775 treatment did not significantly alter cleaved Caspase-3 and cleaved PARP abundance in 105C day-3 spheroid cells (Figure 6f,h). The level of cleaved PARP and Caspase-3 appeared to remain consistent throughout the time period of AZD1775 treatment of the 105C spheroids, with an apparent reduction in protein levels 48–72 h after AZD1775 treatment. Overall, AZD1775 has a reduced negative impact on 105C spheroids relative to KOC-7c spheroids in terms of cell viability and the induction of apoptotic markers.

### 3.5. PLK1 as a Potential Marker for Proliferating and Non-Proliferating OCCC Spheroid Cells Linked to Cell Cycle Differences

**Figure 7:**
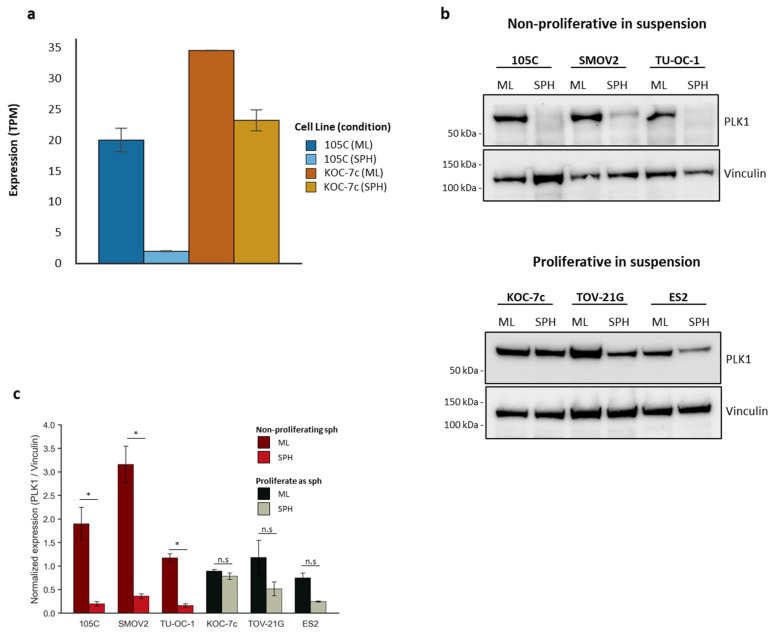
PLK1 expression in OCCC cell lines which proliferate as spheroids and those that do not. (**a**) PLK1 transcript levels (Transcripts Per Million) obtained from RNA-seq showing dramatic downregulation in 105C spheroids. *n* = 2, SEM; (**b**) PLK1 protein levels in OCCC cell lines which do not proliferate as spheroids and OCCC cell lines which can proliferate in spheroid form. Vinculin used as loading control. (**c**) Bars represent relative protein expression (PLK1/Vinculin, arbitrary units) in monolayer (ML) versus spheroid (SPH) cultures for each OCCC cell line (mean ± SD, *n* = 2 paired biological replicates). Paired Student’s *t*-test; ns = not significant, * *p* < 0.05.

PLK1 (polo-like kinase 1) is upstream of CDK1 and plays an essential role in controlling the activity of several enzymes (i.e., CDC25C, CDK1, Wee1) required for the transition from the G2 phase to mitosis [92]. It facilitates the activation of cyclin-dependent kinases (CDKs) and assists in the formation of the mitotic spindle, ensuring proper chromosome alignment and segregation [93,94,95,96,97]. When comparing the response of 105C and KOC-7c cells to spheroid cultures, we find that genes associated with related terms (i.e., “G2M Checkpoint” (GSEA), “Mitotic Spindle” (GSEA), “mitotic cell cycle process” (Metascape Network Analysis), “Chromosome Maintenance” (Enrichr), “Chromosome Condensation” (Enrichr), etc.) are all consistently downregulated in 105C relative to KOC-7c. Additionally, GSEA analysis revealed that “G2M-Checkpoint” and “Mitotic Spindle” are downregulated in 105C spheroid cells relative to KOC-7c. Our RNA-Seq results revealed that *PLK1* (Gene ID: 5347) transcript levels show a drastic decrease in 105C spheroid cells relative to their monolayer and KOC-7c spheroids (Figure 7a). To determine whether this translated to a reduction in PLK1 protein levels, drawing from OCCC cell lines which proliferate as spheroids and those that do not, we performed Western blot analyses using lysates from spheroid and monolayer cells (Figure 7b). We observed that while both 105C and KOC-7c show easily detectable levels of PLK1 protein in monolayer cells, the OCCC cell lines which do not proliferate as spheroids exhibit a dramatic reduction in PLK1, while those that can grow in spheroid form do not show an appreciable downregulation of PLK1 protein. These results further support our finding that non-proliferating OCCC cell line spheroids are in quiescent states and downregulate key regulators of the G2/M transition.

## 4. Discussion

Our goal here was to reveal the transcriptomic differences between human OCCC cells that will undergo dormancy in 3D spheroid form and those that do not as a way of revealing potential drug-specific vulnerabilities. It is now well established that 3D clusters are abundant in the ascites of late-stage epithelial ovarian cancer patients and that these structures mediate the spread of disease throughout the peritoneal cavity. There are reports that these clusters are composed of proliferating cells or a combination of dormant and proliferating cells [50,71,98,99,100]. We focused on two OCCC lines, 105C and KOC-7c, which display similar gene mutation profiles, often detected in OCCC tumors, namely hyperactivated AKT signaling and *ARID1A* truncating mutations. We previously reported that the KOC-7c line proliferates in 3D spheroid form whereas the 105C cell line does not, instead forming spheroids which become dormant but will reactivate the cell cycle upon reattachment to an adhesive substratum [60]. Here, we provide new insights into how ovarian clear cell carcinoma (OCCC) cells adapt to non-adherent spheroid conditions that mimic aspects of peritoneal dissemination. Despite its clinical relevance, OCCC’s distinctive biology, particularly its ability to form and sustain ascites-associated spheroids, remains incompletely understood, limiting the development of targeted therapies. By comparing two OCCC cell lines with contrasting proliferative behaviors in spheroids, our work addresses a significant gap in understanding how these cells sustain or suspend growth under anchorage-independent conditions, and how these adaptations shape therapeutic responsiveness. To our knowledge, this is the first time that the transcriptomes of spheroid cells have been compared to their monolayer counterparts and between lines to elucidate pathways unique to each phenotype and culture condition.

Our findings reveal that OCCC cells adopt divergent molecular strategies to navigate the transition to spheroid culture. KOC-7c cells maintain an active cell cycle program in suspension, sustaining the expression of key mitotic regulators such as PLK1, CDC25C, and CDK1. This ongoing proliferation, even in the absence of anchorage, reflects a robust regulatory network capable of overriding canonical adherence-dependent cell cycle checkpoints. In contrast, 105C spheroids downregulate these same regulators, most notably components of the G2/M checkpoint, including *PLK1*, *Wee1*, *CDC25C,* and *CDK1,* leading to a quiescent or dormant-like state. We extended those studies to other OCCC cell lines to determine whether the results were generally applicable to OCCC cell lines with similar morphologies. With regard to Wee1, CDC25C, and pCDK1 levels in 10 OCCC cell lines, we showed that in the non-proliferative spheroids, the level of those cell cycle regulatory factors dropped dramatically in the dormant spheroids relative to the growing monolayer culture counterparts. This matched what we determined using the 105C cell line. In contrast, those OCCC cell lines which proliferate as spheroids (TOV-21G, RMG-I and ES2) replicated the data we obtained with the KOC-7c cell line, where steady-state levels of the above G2/M regulators remained at levels like that of a growing monolayer of cells. Mechanistically, these transcriptional shifts are mirrored at the protein level, with KOC-7c spheroids retaining sufficient levels of PLK1 and activating phosphatases to progress through mitosis, while 105C spheroids reduce both transcript and protein abundance of these critical regulators. This differential regulation effectively stalls 105C cells at the G2/M boundary, limiting susceptibility to agents targeting mitotic progression.

The central role of PLK1 in regulating proliferative spheroid behavior further underscores its potential as both a biomarker and therapeutic target in OCCC. PLK1 expression was robustly maintained in KOC-7c spheroids but significantly diminished in dormant 105C spheroids, aligning with their divergent cell cycle profiles. These trends were also observed in the SMOV-2 and TU-OC-1 cell lines, which form dormant spheroids and display a loss of PLK1 in spheroids relative to monolayer cells. The TOV-21G and ES2 spheroids, like the KOC-7c cell line, showed easily detectable levels of PLK1 in spheroids, similar to the steady-state level present in growing monolayer cultures. These findings suggest that PLK1-dependent pathways are important for sustaining proliferation in suspension.

Our RNA-Seq analyses revealed distinct transcriptomic profiles between proliferating monolayer 105C cells and their spheroid counterparts, with numerous genes differentially regulated due to 3D spheroid culture. This was also most evident from our PCA plot where 105C monolayer and spheroid cells exhibited the largest variance between culture conditions. Notably, the several pathways related to enhanced viability under non-proliferative conditions were activated in 105C spheroids relative to KOC-7c spheroid cells. mTORC signaling-, hypoxia-, and glycolysis-related gene programs were significantly and specifically elevated in dormant 105C spheroids. Surprisingly, the Enrichr analyses showed elevated translational/protein signatures specifically in 105C spheroid cells relative to both monolayer and proliferating KOC-7c spheroid cells. Perhaps the dormant phenotype induced in day-3 105C spheroid cells requires relatively enhanced translational capacity to maintain that phenotype, as seen by others [101,102,103,104].

The distinct phenotypes of KOC-7c and 105C spheroids also provide a mechanistic basis for their differential sensitivity to Wee1 inhibition. Wee1 kinase enforces the G2/M checkpoint by phosphorylating and inhibiting CDK1, preventing premature mitotic entry. Wee1 inhibitors, such as AZD1775, drive cells prematurely into mitosis, leading to DNA damage and eventual cell death. Others have reported AZD1775 EC50 values do not differ substantially between cell lines generated from different cancer sites [105,106,107,108,109]. We observed the same EC50 values ranging from ~300 nM to 900 nM in monolayer OCCC cells; however, notable differences were revealed when OCCC lines were maintained as spheroids. This effect was prominent in proliferative KOC-7c spheroids, where sufficient levels of CDK1 and PLK1 supported the cytotoxic activity of AZD1775. We included the TOV-21G and JHOC-5 cell lines along with KOC-7c in the AZD1775 EC50 determinations to understand whether the trends carried across to other OCCC cell lines with a similar spheroid phenotype. Indeed, we observed a relatively small change in EC50 compared to the monolayer counterpart cultures since cells from these lines proliferate under both culture conditions. However, in dormant 105C spheroids, where CDK1 and other mitotic regulators are significantly downregulated, Wee1 inhibition failed to trigger lethal mitotic events. We included the OVMANA and RMG-V cell lines which generate dormant spheroids like the 105C cell line and they, similarly, exhibited increased AZD1775 EC50 values relative to their monolayer cultures. Therefore, our observations imply that the efficacy of agents which target cell cycle regulators is best against spheroids in which the cells remain proliferative. Thus, the therapeutic efficacy of Wee1 inhibitors depends on the presence of an intact and active G2/M checkpoint, a feature absent in the quiescent spheroid state. These findings highlight dormancy as a protective state that insulates cells from therapies targeting active cell cycle progression.

The observation that certain cell lines grown in suspension are resistant to AZD1775 relative to the monolayer might be expected given the function of the Wee1 kinase, as a key G2/M checkpoint regulator. However, our observation that the KOC-7c, JHOC-5, and TOV-21G showed an increased sensitivity compared to their monolayer counterparts is somewhat surprising. This could be due to the added stress of anchorage-independent growth which may enhance the effect of Wee1 inhibition on those spheroid cells. This observation indicates that the sensitivity to AZD1775 may be influenced not solely by proliferation rate but also by alterations in signaling pathways, stress responses, or metabolic dependencies that are uniquely activated in spheroids as we found through our Enrichr analysis of the RNA-Seq.

Beyond cell cycle regulation, the biological differences between 105C and KOC-7c spheroids extend to metabolic and stress response pathways. While both cell lines exhibit some common transcriptional adjustments under spheroid conditions, such as altered metabolic programs, 105C spheroids appear to pivot toward survival-oriented programs, including autophagy and ribosomal modulation. These adaptations likely conserve energy and sustain cellular integrity during dormancy, allowing cells to survive unfavorable conditions. Conversely, KOC-7c spheroids leverage metabolic reprogramming to support proliferation, enhancing their metastatic potential and resistance to certain therapies. Interestingly, AKT signaling is hyperactivated in 105C and KOC-7c cells due to the presence of specific gene mutations [60] and this hyperactivation is not changed by spheroid culture even though 105C spheroid cells are dormant. It is well established that constitutively active AKT signaling leads to elevated protein synthesis and translational capacity through mTOR-containing complexes [110,111,112,113,114]. Moreover, enhanced AKT signaling would be expected to have a negative regulatory effect on Wee1 kinase activity through inhibitory phosphorylation [115]. Therefore, our data suggests that, despite a nutrient-rich environment and activated AKT signaling, spheroid formation induces a dormant phenotype in 105C cells by downregulating cell cycle regulators of G2/M transition. These distinct survival strategies underscore the heterogeneity within OCCC and emphasize the need for personalized therapeutic approaches based on a tumor’s molecular state.

From a translational perspective, our findings suggest that several therapeutic strategies are available for managing OCCC. For tumors resembling the KOC-7c phenotype, characterized by active cycling even in suspension, treatments targeting the G2/M checkpoint, such as Wee1 or PLK1 inhibitors, may prove particularly effective. Conversely, for tumors with a dormant, 105C-like spheroid phenotype, alternative approaches are needed. These could include agents that disrupt dormancy pathways, reawaken quiescent cells, or target the metabolic and stress response circuits essential for long-term spheroid survival [101,116,117,118]. Combining dormancy-disrupting agents with cell cycle inhibitors may further enhance therapeutic efficacy by sensitizing previously dormant cells to checkpoint-targeted therapies.

We acknowledge some limitations of this study. Our experiments were conducted in vitro using established cell lines, which may not fully recapitulate the complexity of the tumor microenvironment. Future studies should investigate these mechanisms in patient-derived organoids, co-cultures with stromal and immune cells, and in vivo models to validate their clinical relevance. Additionally, exploring the roles of epigenetic regulation, hypoxia-driven pathways, and extracellular matrix components could provide a more comprehensive understanding of how OCCC spheroids arise, persist, and respond to therapy.

In conclusion, this study advances our understanding of how OCCC cells adapt to suspension by either sustaining or suspending cell cycle progression. By linking transcriptional and protein-level changes in key regulators such as PLK1 and Wee1 to differential drug sensitivities, we illuminate critical vulnerabilities and resistance mechanisms within OCCC spheroids. These findings not only address fundamental questions about OCCC biology but also pave the way for more refined therapeutic strategies aimed at disrupting the spheroid-mediated survival strategies that underlie metastasis and recurrence.

## Figures and Tables

**Table 1:** Antibodies used in immunoblotting experiments.

Antibody	Company	Catalogue #
Caspase-3	Cell Signaling Technology (Danvers, MA, USA)	9662
Caspase-3 (cleaved)	Cell Signaling Technology	9661
CDK1	Cell Signaling Technology	9116
CDC25C	Cell Signaling Technology	4688
γH2AX	Cell Signaling Technology	9718
p-CDK1 (Tyr15)	Cell Signaling Technology	4539
p-Histone H3 (Ser10)	Cell Signaling Technology	9701
Wee1	Cell Signaling Technology	13084
PARP (cleaved)	Cell Signaling Technology	9541
PLK1	Cell Signaling Technology	4513S
Vinculin	MilliporeSigma (Burlington, MA, USA)	V9264
Actin	MilliporeSigma	A2066
Anti-rabbit IgG	MilliporeSigma	NA934V
Anti-mouse IgG	MilliporeSigma	NA931V

## Data Availability

The data discussed in this publication have been deposited in NCBI’s Gene Expression Omnibus and are accessible through GEO Series accession number GSE294002.

## Supplementary Materials

The following supporting information can be downloaded at: https://www.mdpi.com/article/10.3390/cells14110785/s1, Figure S1. Fold change of genes responsible for “Translational/Protein Synthesis Signature” being up in 105C SPH/ML vs KOC-7c SPH/ML; Figure S2. siRNA-mediated knockdown of CDK1 in KOC-7c and JHOC5(LT66) cells; Figure S3. JHOC-5 spheroid growth curve. Figure S4. Biological replicates of Western blots used in Figure 4; Figure S5. Effects of AZD1775 EC50 treatment on 105C monolayer cells. Table S1. Differentially expressed genes (ML vs SPH) in 105C and KOC-7c cell lines; Table S2. Differentially expressed genes between cell lines 105C (ML vs SPH) vs KOC-7c (ML vs SPH); Table S3. EC50 values for AZD1775 in OCCC cell lines (related to Figure 5b).

## Abbreviations

The following abbreviations are used in this manuscript:

DMEM	Dulbecco’s Modified Eagle Medium
DMSO	Dimethyl Sulfoxide
EC50	Half Maximal Effective Concentration
EC95	Concentration producing 95% of maximal effect
EOC	Epithelial ovarian cancer
FBS	Fetal Bovine Serum
GSEA	gene set enrichment analysis
ML	Monolayer
NES	Normalized Enrichment Score
OCCC	Ovarian Clear Cell Carcinoma
PBS	Phosphate-Buffered Saline
PCA	Principal Component Analysis
pH3	Phospho-Histone H3 (Ser10)
RIPA	Radioimmunoprecipitation Assay
RNA-Seq	RNA Sequencing
SPH	Spheroid
TBST	Tris-Buffered Saline containing Tween-20
ULA	ultralow attachment
