# Supplementary material for: Transcriptomic Analyses of Ovarian Clear Cell Carcinoma Spheroids Reveal Distinct Proliferative Phenotypes and Therapeutic Vulnerabilities
Source: Cells. 2025 May 27;14(11):785. doi: 10.3390/cells14110785 (PMC12154277; doi:10.3390/cells14110785)
Supplement: Supplementary file 1 [file cells-14-00785-s001.zip › Figure S1. Fold-change of genes responsible for Translational Protein Synthesis Signature.pdf]

Genes responsible for “Translational / Protein Synthesis Signature” being UP in 105C SPH/ML vs KOC7C SPH/ML

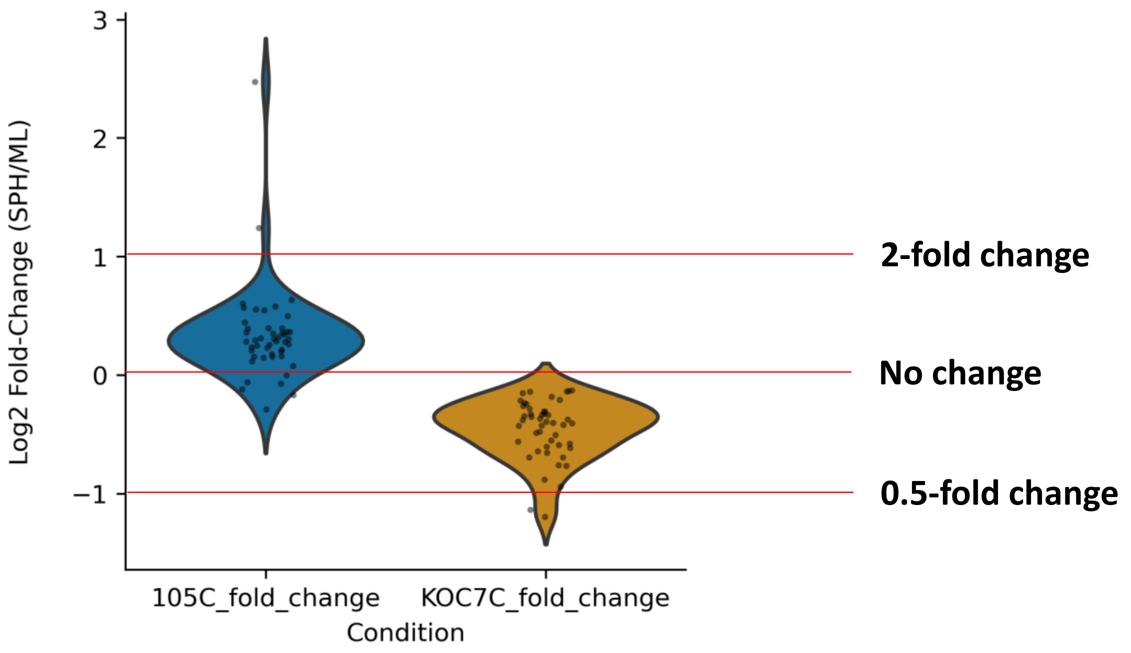

Figure S1. Fold-change of genes responsible for “Translational / Protein Synthesis Signature” being up in 105C SPH/ML vs KOC7C SPH/ML. RNA-Seq expression data was used to observe the change in expression (SPH/ML) of genes responsible for the ‘Translation / Protein synthesis signature’ being up in 105C vs KOC-7c. We note that a slight elevation in these genes occurs in 105C (SPH/ML) while a downregulation occurs in the KOC-7c
