# Supplementary material for: Transcriptomic Analyses of Ovarian Clear Cell Carcinoma Spheroids Reveal Distinct Proliferative Phenotypes and Therapeutic Vulnerabilities
Source: Cells. 2025 May 27;14(11):785. doi: 10.3390/cells14110785 (PMC12154277; doi:10.3390/cells14110785)
Supplement: Supplementary file 1 [file cells-14-00785-s001.zip › Figure S2. siRNA-mediated knockdown of CDK1 in KOC-7c and JHOC5(LT66) cells.pdf]

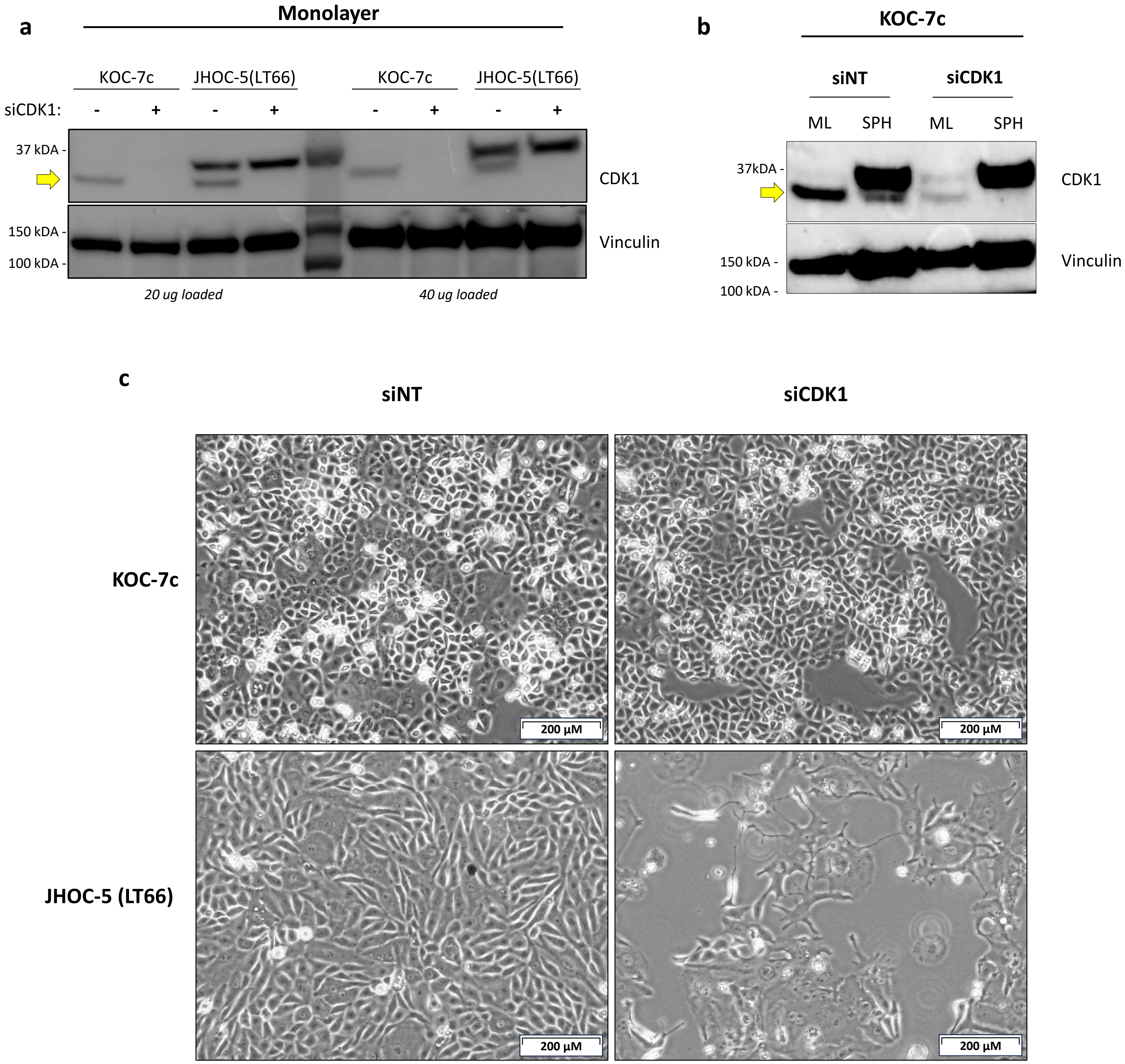

Figure S2. siRNA-mediated knockdown of CDK1 in KOC-7c and JHOC5(LT66) cells. (**a**) KOC-7c and JHOC-5(LT66) cells grown in monolayer were transfected with either scrambled (siNT) or CDK1-targeting siRNA (siCDK1). At 72 h post-transfection cell lysates were prepared and analyzed by SDS-PAGE followed by immunoblotting with an anti-CDK1 antibody; Vinculin was used as a loading control; (**b**) KOC-7c cells were transfected as in (A) then split into monolayer (ML) or suspension (SPH) cultures. After 72 h in each condition cell lysates were collected and probed for CDK1 and Vinculin as above. Yellow arrows shows the CDK1 band is lost after siCDK1 knockdown while the upper non-specific band remains; (**c**) Representative Brightfield microscopy images of KOC-7c and JHOC-5(LT66) cells at 72 h post-transfection. While KOC-7c cells showed no marked morphological change compared with scrambled controls (siNT), JHOC-5(LT66) cells displayed pronounced morphological alterations including reduced confluency and rounding suggestive of decreased viability and senescence.
