# Supplementary material for: Transcriptomic Analyses of Ovarian Clear Cell Carcinoma Spheroids Reveal Distinct Proliferative Phenotypes and Therapeutic Vulnerabilities
Source: Cells. 2025 May 27;14(11):785. doi: 10.3390/cells14110785 (PMC12154277; doi:10.3390/cells14110785)
Supplement: Supplementary file 1 [file cells-14-00785-s001.zip › Figure S3. Growth of JHOC-5 in long-term spheroid suspension culture.pdf]

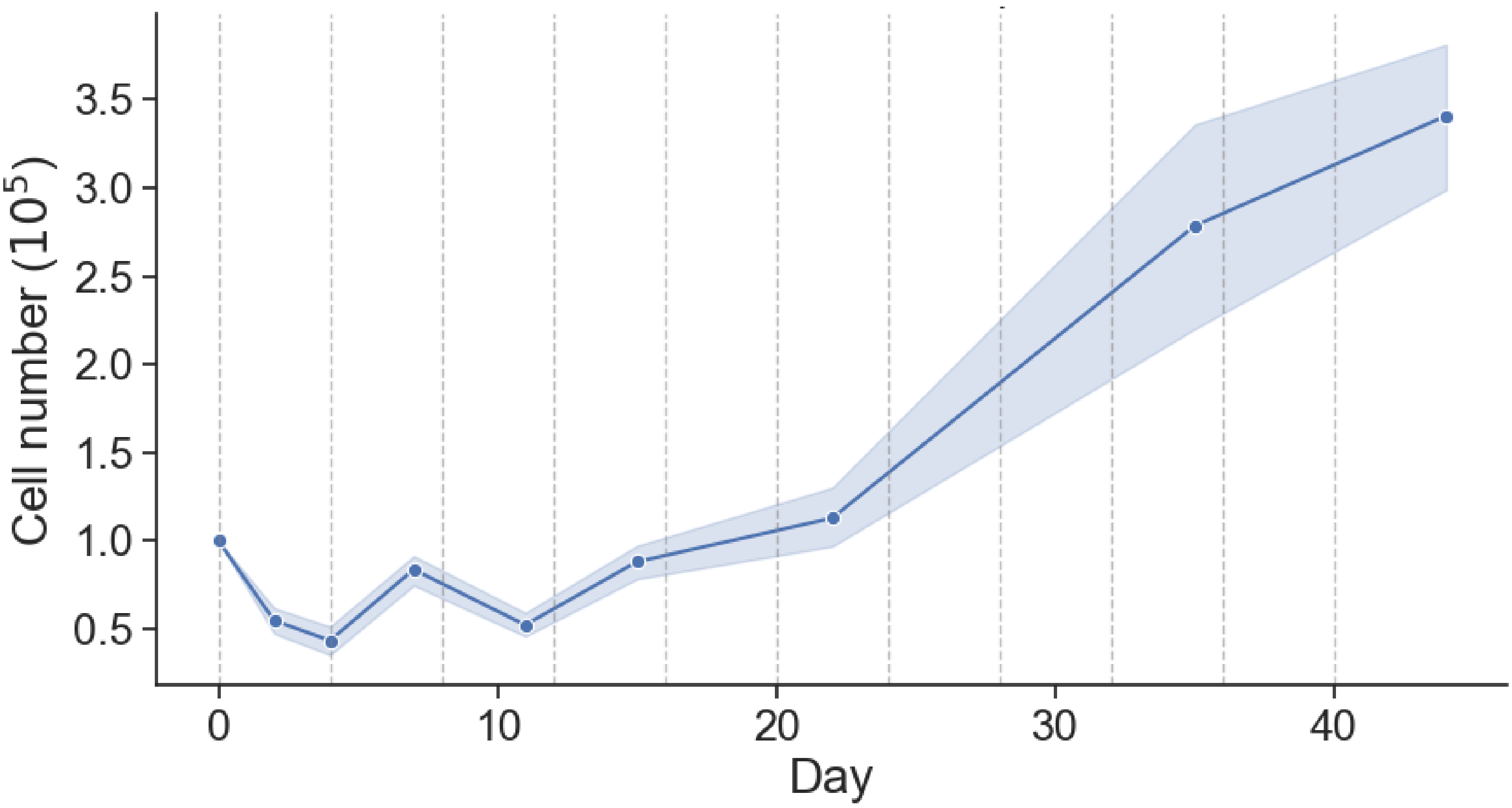

Figure S3. Growth of JHOC-5 in long-term spheroid suspension culture. The JHOC-5 was seeded in ultra low attachment 24-well plates at a density of 100,000/well (day 0). The number of live cells was determined at semi-regular intervals over the duration of the experiment (filled circles) and media was replaced every 4 days (dashed vertical lines). The wide-band following each line indicates -/+ standard error of the mean (n=6).
