# Supplementary material for: Transcriptomic Analyses of Ovarian Clear Cell Carcinoma Spheroids Reveal Distinct Proliferative Phenotypes and Therapeutic Vulnerabilities
Source: Cells. 2025 May 27;14(11):785. doi: 10.3390/cells14110785 (PMC12154277; doi:10.3390/cells14110785)
Supplement: Supplementary file 1 [file cells-14-00785-s001.zip › Figure S5. Effects of AZD1775 EC50 treatment on 105C monolayer cells.pdf]

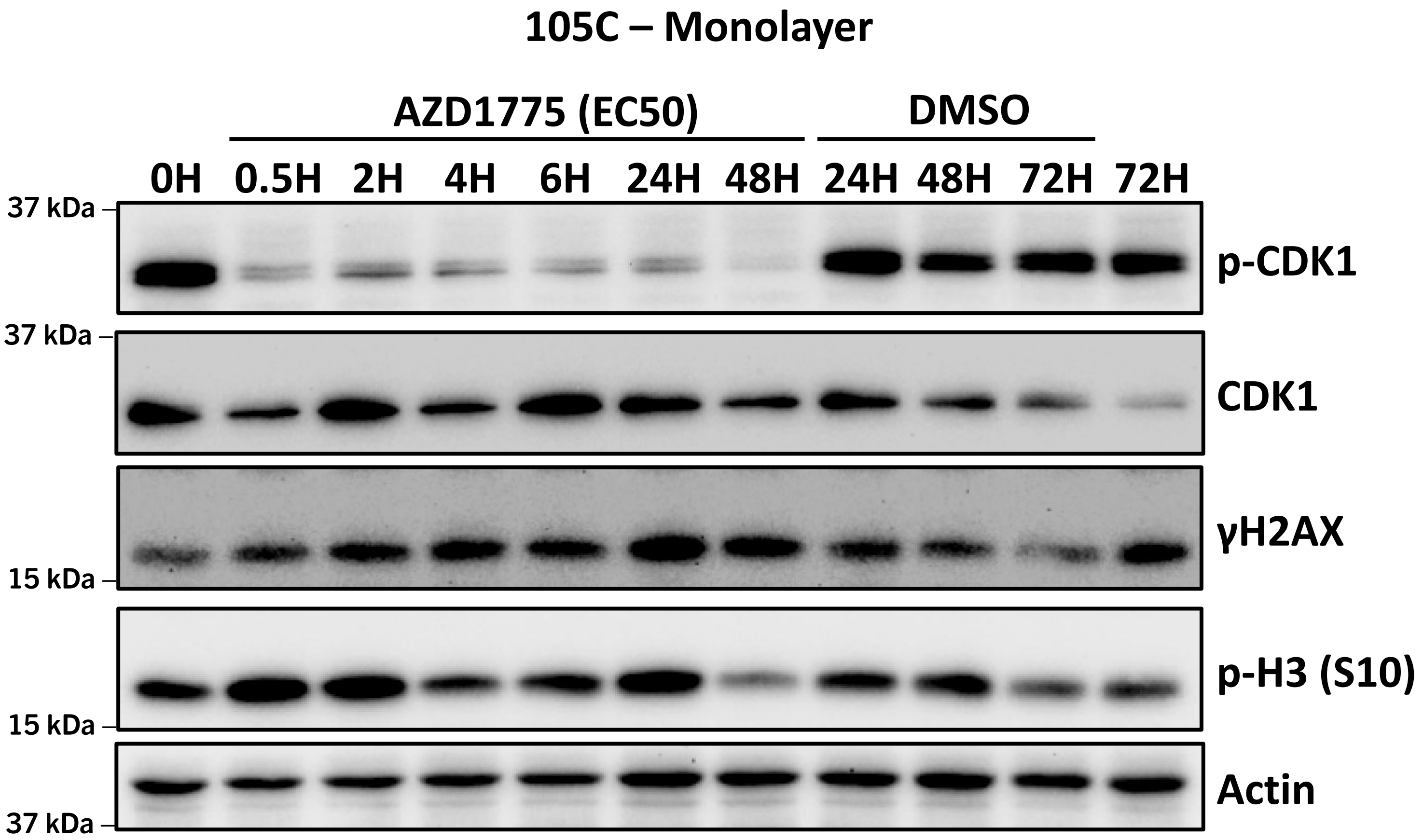

Figure S5. Effects of AZD1775 EC50 treatment on 105C monolayer cells. Cells were seeded in 6-well adherent culture plates at densities that yielded ~90 % confluency at time of lysate collection. Cells were treated 3 days post-seeding with their monolayer EC50 concentration (250 nM) and whole-cell protein lysates were collected at every time point and used for western blot analysis for phospho-CDK1 (P-CDK1), total CDK1, γH2AX, and phospho-H3 (P-H3). Actin was used as loading control.
