# Supplementary material for: Transcriptomic Analyses of Ovarian Clear Cell Carcinoma Spheroids Reveal Distinct Proliferative Phenotypes and Therapeutic Vulnerabilities
Source: Cells. 2025 May 27;14(11):785. doi: 10.3390/cells14110785 (PMC12154277; doi:10.3390/cells14110785)
Supplement: Supplementary file 1 [file cells-14-00785-s001.zip › Table S3. EC50 values for AZD1775 in OCCC cell lines (related to Figure 5b).pdf]

Table S3. EC50 values for AZD1775 in OCCC cell lines (related to Figure 5b)

**Cell line**	**Culture Condition**	**EC50 (nanomolar)**
105C	ML	267.86
105C	SPH	2915.79
OVMANA	ML	276.61
OVMANA	SPH	789.59
RMG-V	ML	309.28
RMG-V	SPH	927.56
KOC-7c	ML	961.37
KOC-7c	SPH	605.13
TOV-21G	ML	561.26
TOV-21G	SPH	171.62
JHOC-5	ML	502.82
JHOC-5	SPH	239.07
