# Supplementary figures and images for: Transcriptomic Analyses of Ovarian Clear Cell Carcinoma Spheroids Reveal Distinct Proliferative Phenotypes and Therapeutic Vulnerabilities
Source: Cells. 2025 May 27;14(11):785. doi: 10.3390/cells14110785 (PMC12154277; doi:10.3390/cells14110785)

**a**

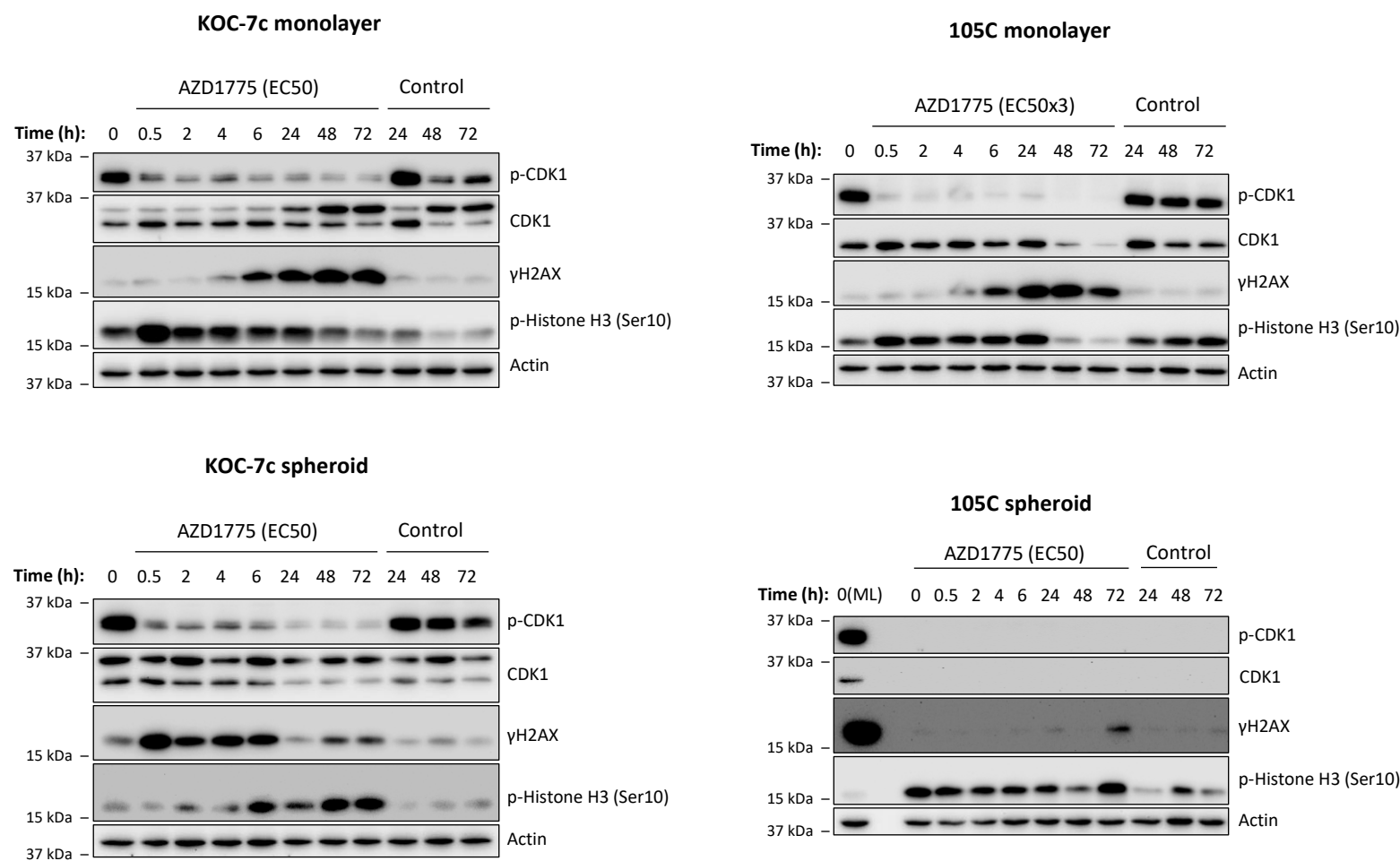

**Figure S4.** Biological replicates of western blots used in Figure 4.

Supplement: Supplementary file 1 [file cells-14-00785-s001.zip › Figure S4. Biological replicates of western blots used in Figure 4.pdf]
